# Supplementary figures and images for: The neurohormone tyramine stimulates the secretion of an insulin-like peptide from the Caenorhabditis elegans intestine to modulate the systemic stress response
Source: PLoS Biol. 2025 Jan 28;23(1):e3002997. doi: 10.1371/journal.pbio.3002997 (PMC11774402; doi:10.1371/journal.pbio.3002997)

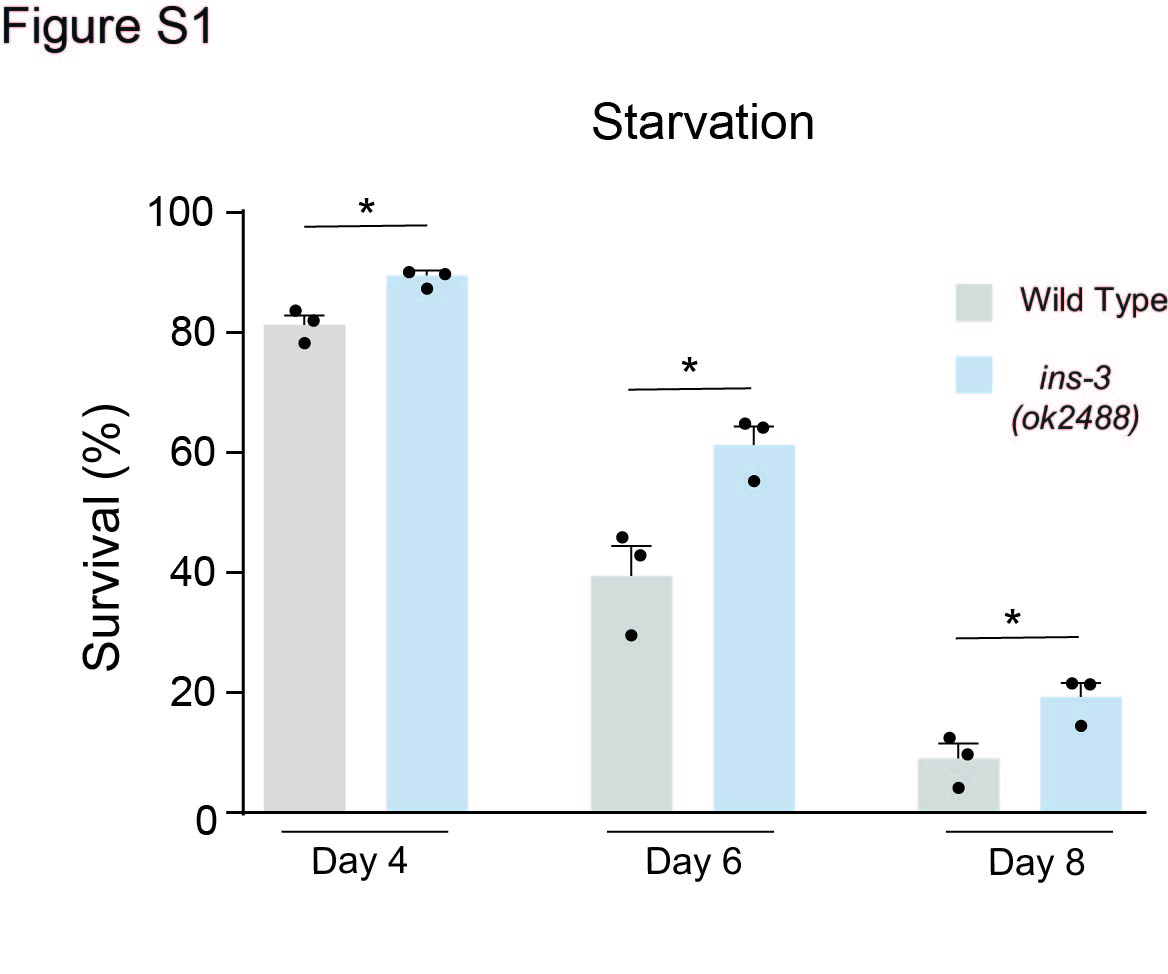

Supplement: S1 Fig — Survival percentages of wild-type and ins-3 null mutant worms upon 4, 6, and 8 days of starvation. Three independent experiments were performed n = 3. Each experiment included 45–50 animals per condition. Two-tailed Student’s t test was used. * p < 0.05. The data underlying this figure can be found at https://osf.io/wfgvs/. (JPG) [file pbio.3002997.s001.jpg]

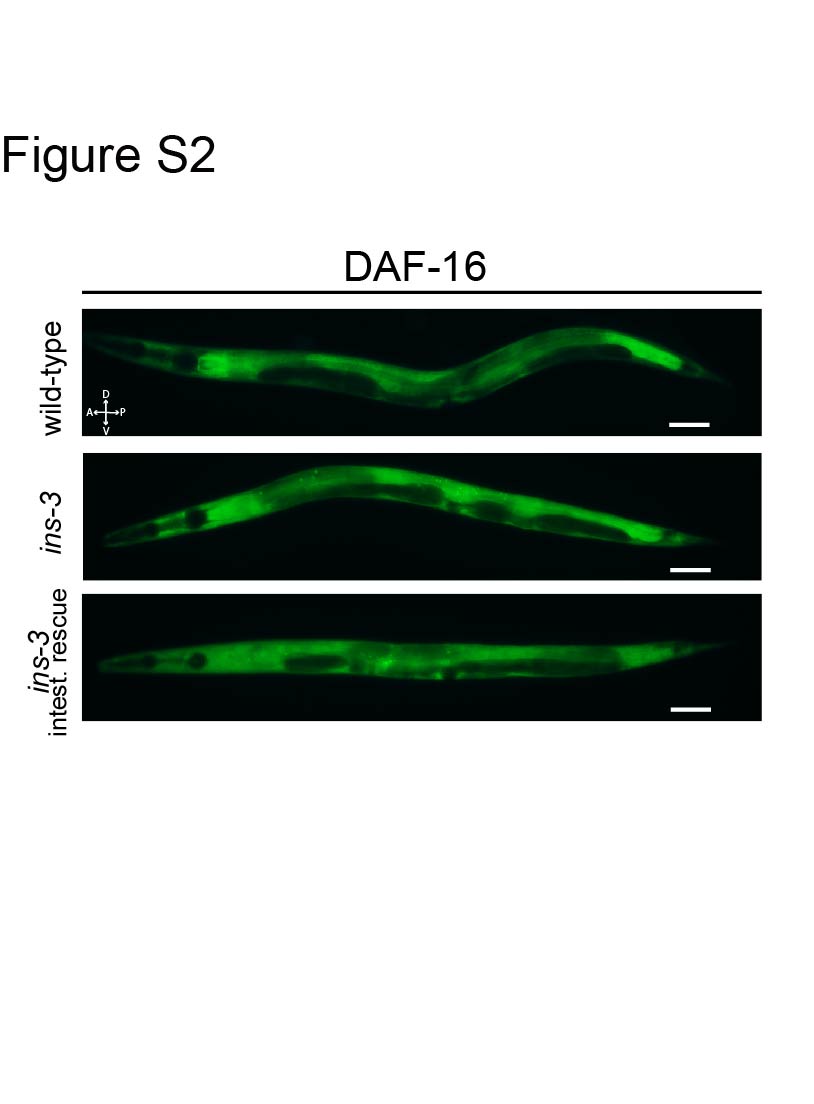

Supplement: S2 Fig — Representative fluorescence images (20×) depicting the localization of DAF-16a/b::GFP under basal conditions in wild-type, ins-3 null mutant, and intestinal rescue of ins-3 backgrounds. No nuclear localization of DAF-16 was observed. n = 20–30 animals per condition. Scale bar, 50 μm. (JPG) [file pbio.3002997.s002.jpg]

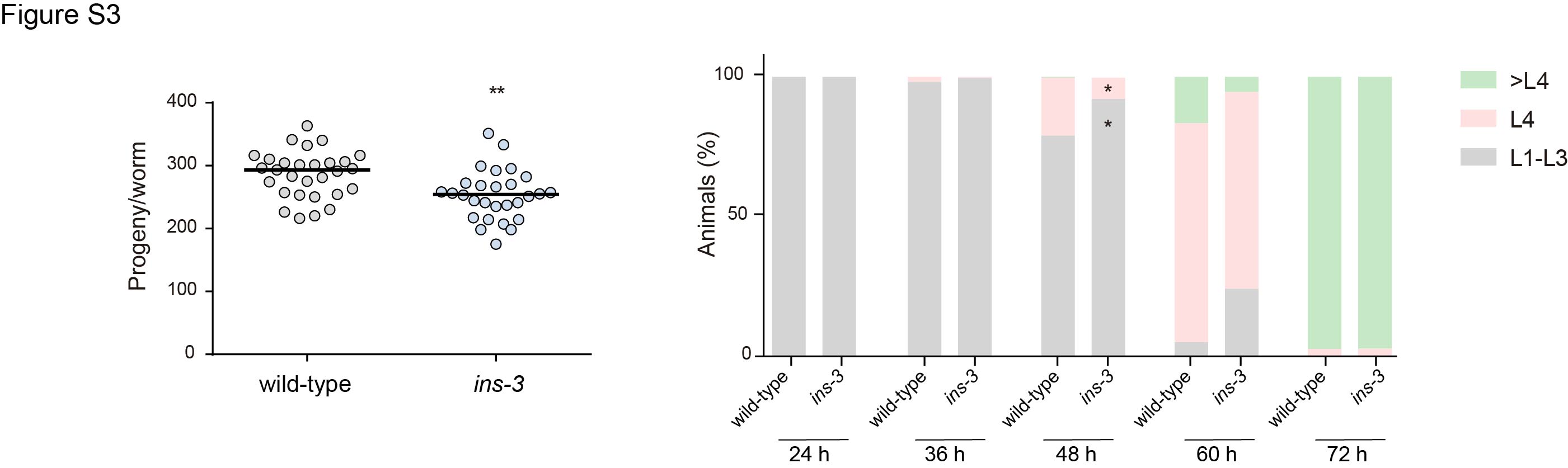

Supplement: S3 Fig — Left. Total number of progeny per worm in wild-type and ins-3 null mutants. n = 25–30 animals per condition distributed across (three) independent experiments. Two-tailed Student’s t test was used. **p < 0.01. Right. Developmental rate of wild-type and ins-3 null mutant worms. A color code was used to represent each of the following animal stages: L1–L3: early larval stages, L4: last larval stage, > L4: adult stage. The animal classification was evaluated at the indicated time points (24, 36, 48, 60, and 72 h). Five to six independent experiments were performed (n = 5–6). Each experiment included 100–200 worms per condition Data are represented in a stacked bar chart as mean. A two-tailed Student’s t test was used. * p < 0.05. The data underlying this figure can be found at https://osf.io/wfgvs/. (JPG) [file pbio.3002997.s003.jpg]

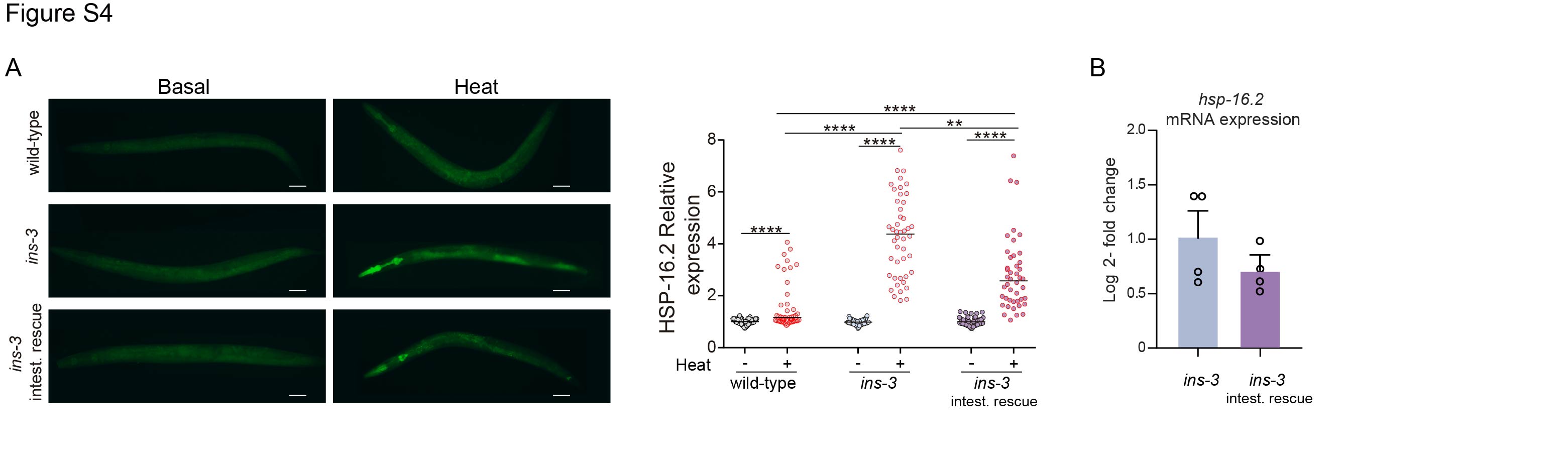

Supplement: S4 Fig — (A) Representative fluorescence images (20×) of animals expressing Phsp16.2::GFP in wild-type, ins-3 null mutants, and intestinal rescue of ins-3 backgrounds under basal conditions and after 15 min of heat exposure (35 °C) followed by 70 min recovery at 20 °C. Scale bar, 50 μm. Right. Corresponding quantification of the fluorescence level per animal. Scatter dot plot with relative expression of Phsp16.2::GFP (normalized to wild type of each independent experiment) (line shows median). n = 10–20 animals per condition distributed across three to four independent experiments. One-way ANOVA and Dunn’s post hoc test for multiple comparisons among groups in basal conditions were used. ns, not significant. One-way ANOVA and Dunn’s post hoc test for multiple comparisons among groups after heat stress were used **p < 0.01, ****p < 0.0001. Two-tailed Student’s t test (basal vs. heat stress) was used. ****p < 0.0001. (B) Log2 fold-changes in hsp-16.2 transcript levels in animals exposed 15 min of heat exposure (35 °C). Negative and positive values indicate down- and up-regulation of this gene compared to wild-type animals, respectively. Fold change was calculated as ΔCt basal conditions/ΔCt test conditions. Results are shown as mean ± s.e.m. The data underlying this figure can be found at https://osf.io/wfgvs/. (JPG) [file pbio.3002997.s004.jpg]

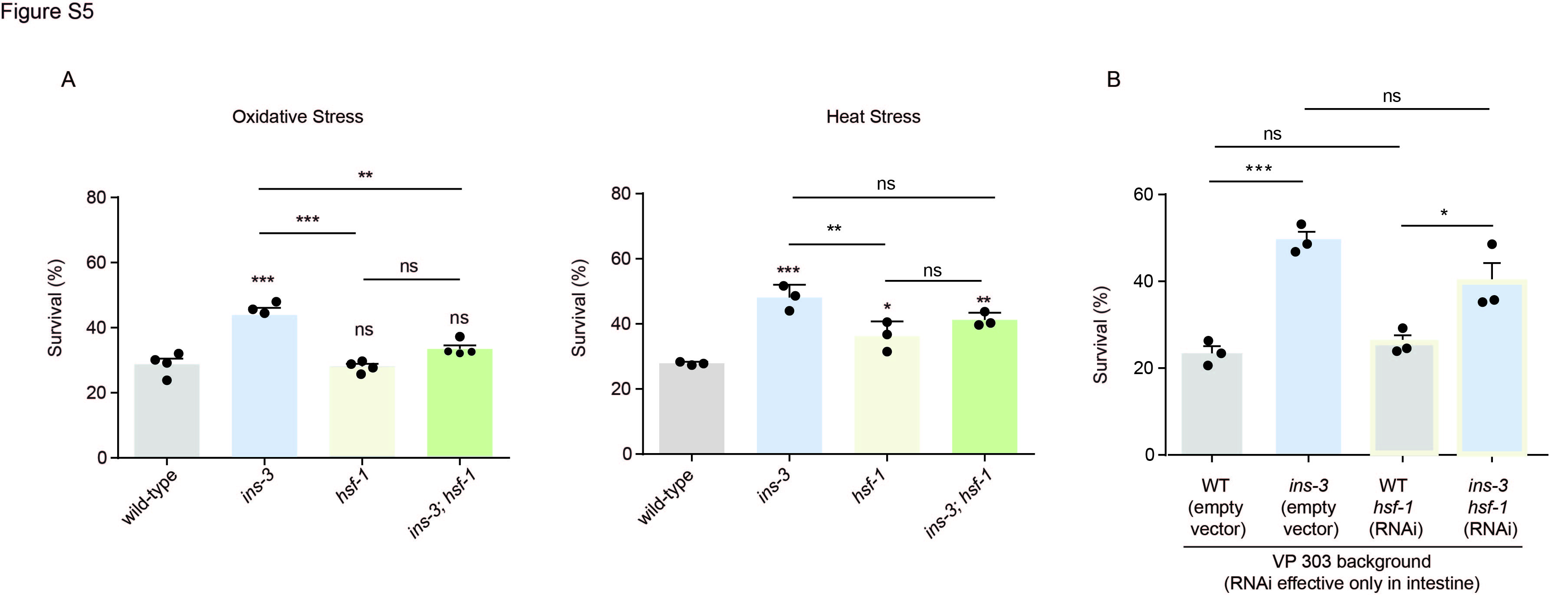

Supplement: S5 Fig — (A) Survival percentages of wild-type animals, ins-3 single mutants, hsf-1 single mutants and ins-3;hsf-1 double mutants exposed to oxidative (left) and heat stress (right). Results are shown as mean ± s.e.m. Five and six independent experiments were performed for oxidative stress and heat, respectively. Each experiment included 50–100 worms per condition. (B) Oxidation resistance of animals subjected to intestinal RNAi-mediated silencing of hsf-1. The VP303 (kbIs7 [nhx-2p::rde-1 + rol-6(su1006)]) background allows RNAi silencing only in the intestine [55,109,110]. RNAi, RNA interference. Results are shown as mean ± s.e.m. Results are shown as mean ± s.e.m. Four independent experiments were performed (n = 4). Each experiment included 30–80 worms per condition. The data underlying this figure can be found at https://osf.io/wfgvs/. (JPG) [file pbio.3002997.s005.jpg]

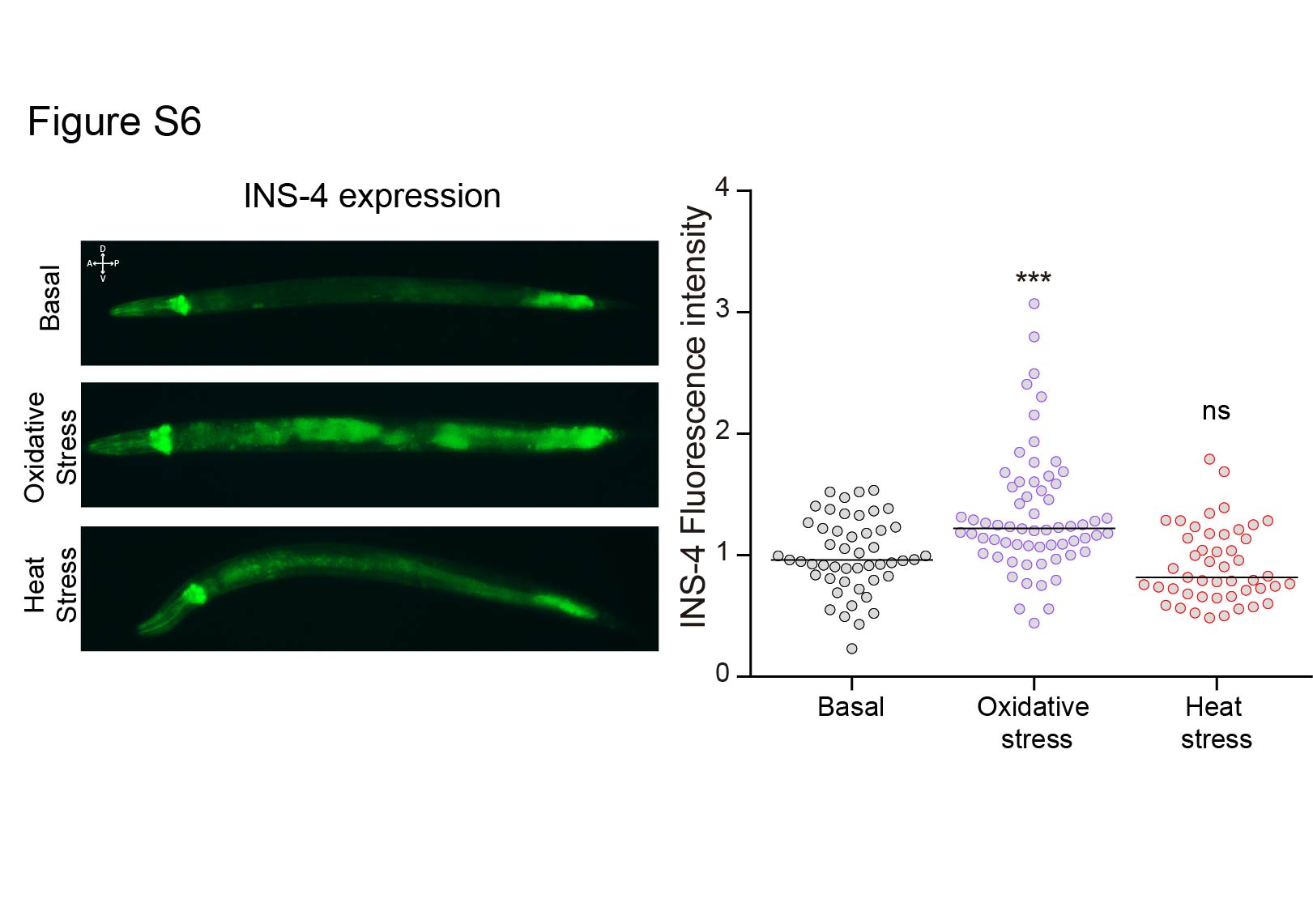

Supplement: S6 Fig — Left. Representative epifluorescence images (20×) of young non-gravid adults expressing Pins-4::GFP under basal conditions, oxidative (2 h, 1 mM FeSO4) or heat stress (6 h, 30 °C). Scale bar, 50 µm. Right. Corresponding quantification of fluorescence levels per worm. Scatter dot plot with relative expression of Pins-4::GFP normalized to the basal condition of each independent experiment. Line at the median. n = 45–60 animals per condition distributed across (three) independent experiments. One-way ANOVA (Kruskal–Wallis test) and Dunn’s post hoc test versus basal were used. ns, not significant, ***p < 0.001. The data underlying this figure can be found at https://osf.io/wfgvs/. (JPG) [file pbio.3002997.s006.jpg]

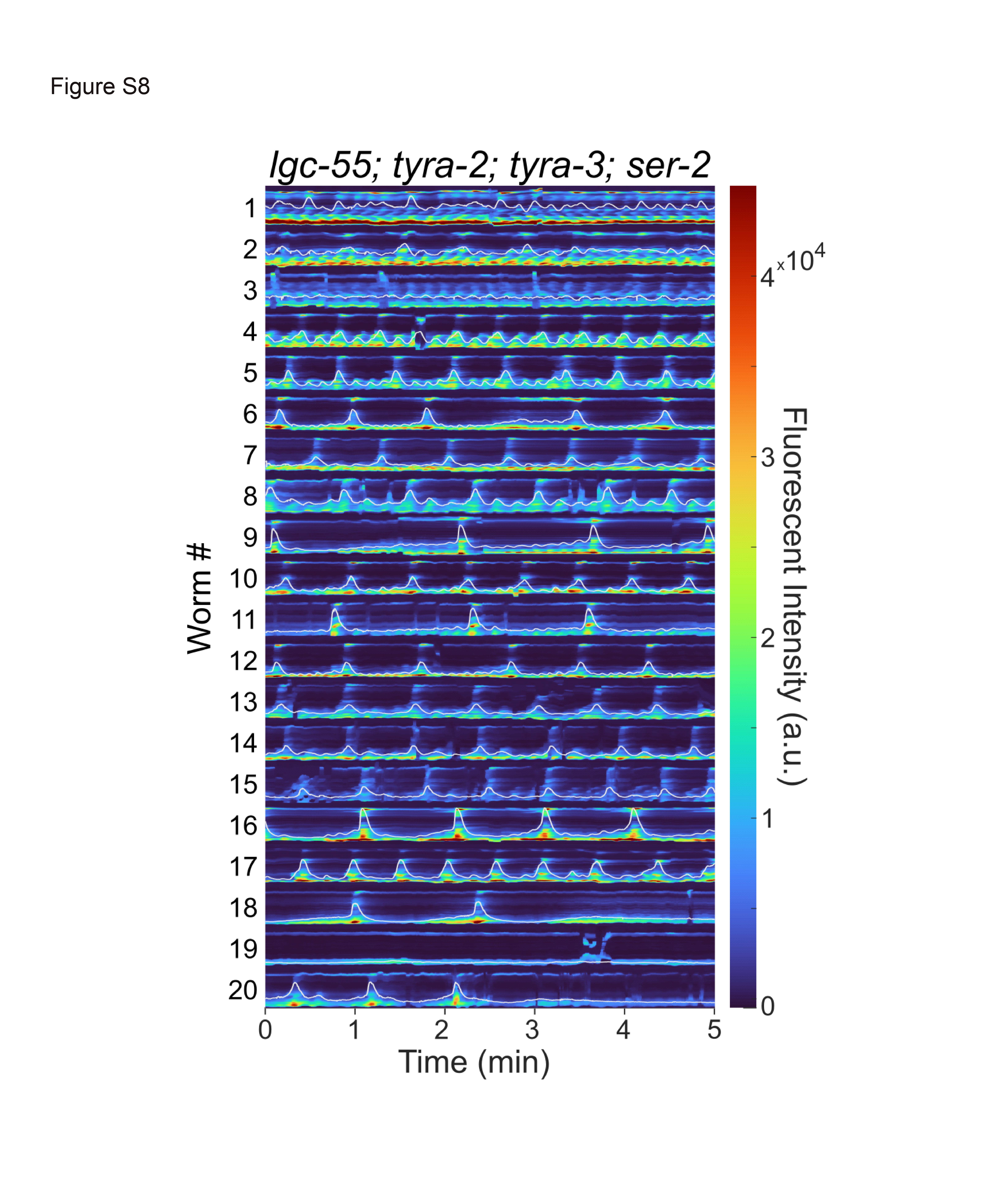

Supplement: S8 Fig — Measurement of intestinal GCaMP fluorescence in lgc-55; ser-2, tyra-2, tyra-3 quadruple mutants exposed to 30 mM tyramine. Kymographs are oriented with the anterior of the animal at the top. The mean fluorescent intensity (white trace) is superimposed on the kymograph with a y-axis range of −1,000–8,000 arbitrary units (a.u.). Colormap values for the kymograph are indicated on the right. Twenty-two animals were recorded in total. The data underlying this figure can be found at https://osf.io/wfgvs/. (JPG) [file pbio.3002997.s008.jpg]

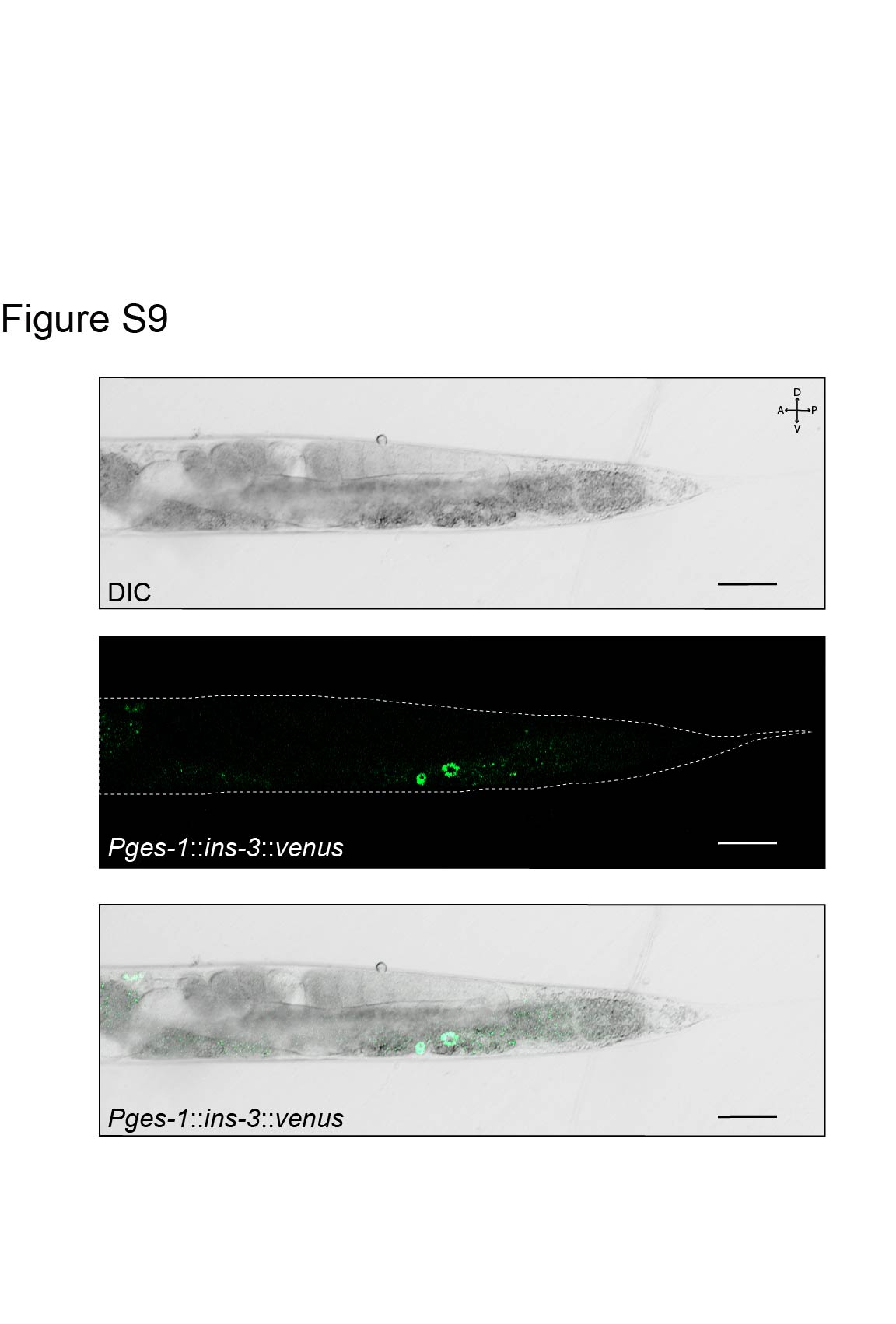

Supplement: S9 Fig — Representative image (40×) of L4-staged worm carrying Pges-1::INS-3::VENUS in basal conditions showed on differential interference contrast (DIC), fluorescence, and merged. Note the fluorescence both in the intestine (faint) and in the coelomocytes. Scale bar, 50 μm. (JPG) [file pbio.3002997.s009.jpg]

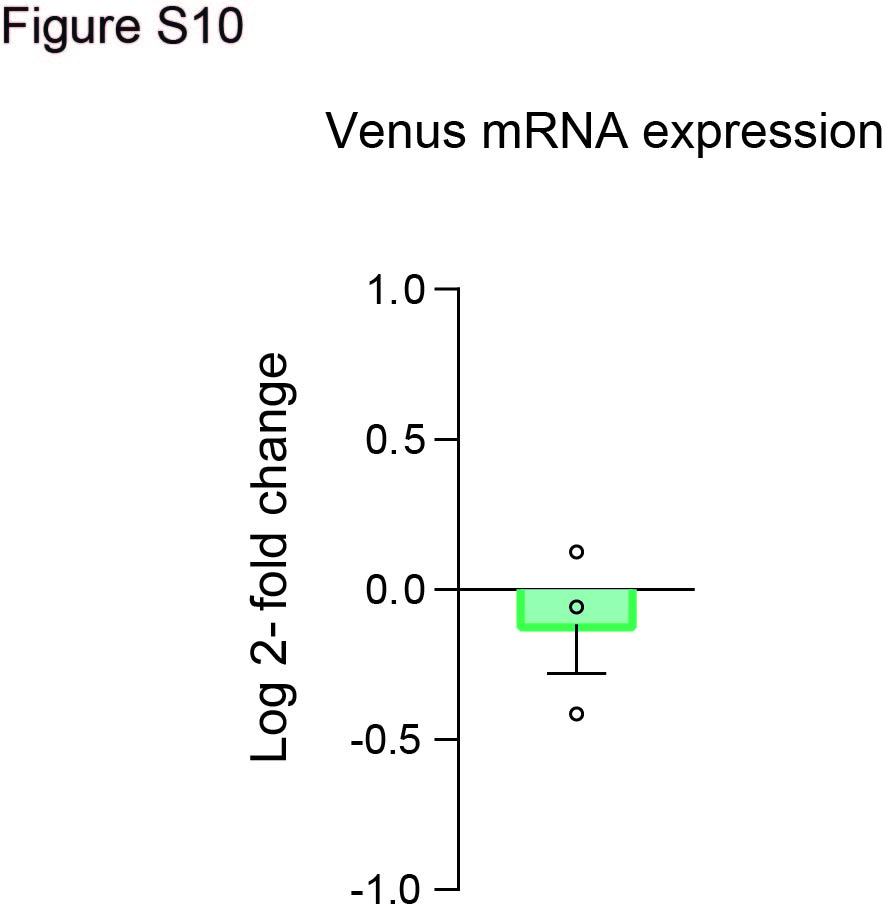

Supplement: S10 Fig — Log2 fold-changes in Venus transcript levels in transgenic animals expressing Pges-1::ins-3::venus exposed to exogenous tyramine (15 mM). Negative and positive values indicate down- and up-regulation of the transcript compared to non-exposed to non-exposed animals. Fold change was calculated as ΔCt no Tyr/ΔCt Tyr. Results are shown as mean ± s.e.m. The data underlying this figure can be found at https://osf.io/wfgvs/. (JPG) [file pbio.3002997.s010.jpg]

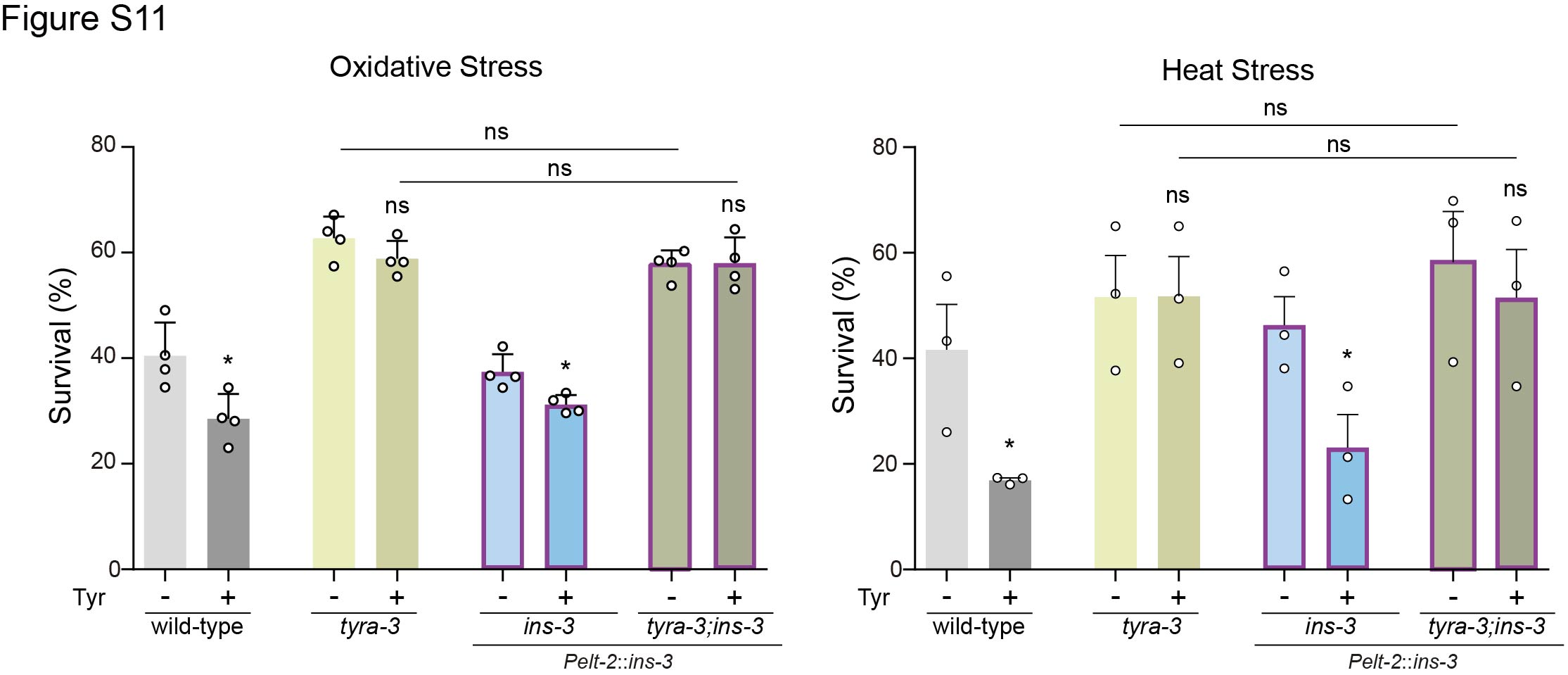

Supplement: S11 Fig — Survival percentages to oxidative (left) and heat stress (right) of wild-type, tyra-3 null mutants, and animals expressing the intestinal rescue of ins-3 on wild-type and tyra-3 null mutant backgrounds in the absence or presence of exogenous tyramine (15 mM), (mean ± s.e.m). Four independent experiments were performed (n = 4). Each experiment included 40−80 animals per condition. For conditions with tyramine, a two-tailed Student’s t test versus the same strain without tyramine was used. ns, not significant, * p < 0.05. Two-tailed Student’s t test was used between tyra-3 null mutants and tyra-3; ins-3; Pelt-2::INS-3 strain. ns, not significant. The data underlying this figure can be found at https://osf.io/wfgvs/. (JPG) [file pbio.3002997.s011.jpg]
