# Supplementary material for: The neurohormone tyramine stimulates the secretion of an insulin-like peptide from the Caenorhabditis elegans intestine to modulate the systemic stress response
Source: PLoS Biol. 2025 Jan 28;23(1):e3002997. doi: 10.1371/journal.pbio.3002997 (PMC11774402; doi:10.1371/journal.pbio.3002997)
Supplement: S1 Table — ΔCT values of ins-3 measured by qPCR in ins-3 null mutants and animals rescued with the Prgef-1::ins-3 transgene. These data support effective neuronal expression of ins-3 in the rescued animals. (PDF) [file pbio.3002997.s017.pdf]

Table S1

	***ins-3* null mutant**		***ins-3; Prgef-1::ins-3* (neuronal rescue)**	
	Ins-3	*actin*	*Ins-3*	*actin*
**CT**	44.53 ± 3.30	14.80 ± 1.44	20.20 ± 1.24	19.33 ± 0.87
**ΔCT (*CT ins-3 - CT actin*)**	29.73 ± 3.52		2.2 ± 1.74	
