# Supplementary material for: The neurohormone tyramine stimulates the secretion of an insulin-like peptide from the Caenorhabditis elegans intestine to modulate the systemic stress response
Source: PLoS Biol. 2025 Jan 28;23(1):e3002997. doi: 10.1371/journal.pbio.3002997 (PMC11774402; doi:10.1371/journal.pbio.3002997)
Supplement: Attachment — Submitted filename: Veutheyetal2024RebuttalPB.pdf [file pbio.3002997.s018.pdf]

We sincerely thank the reviewers for their constructive feedback. In the revised manuscript we have addressed their questions and incorporated their suggestions which have significantly improved our manuscript. We believe that, based on these suggestions, we have strengthened our conclusions with more robust support than in the original version. For this revision, we have added genetic experiments, qPCR analyses, RNAi studies, and calcium imaging, which enhance the significance of our work. These results allow us to conclude more definitively that tyramine modulates the release of the ILP INS-3 in the intestine, and that this release systemically activates DAF-2, inhibiting the ability of *C. elegans* to respond to environmental stressors. Our detailed responses to each of the reviewers' concerns are provided below (in blue). Changes in the revised version are highlighted in yellow:

REVIEWS:

Reviewer #1: Reviewer summary: Veuthey et al. provide evidence to demonstrate the role of INS-3 insulin-like peptide in modulating stress response in C. elegans. They also demonstrated that INS-3 release is induced by tyramine and acts via DAF-2 to modulate the stress response. given the group's prior Nature paper, some more novelty is needed. The following are some issue that needs to be addressed.

Major:
1. Why only oxidative and thermal stress was chosen for the study? What about starvation stress (food-searching behavior or rate of motility) and introduction to pathogenic bacterial strains (percentage survival or reduced lifespan)? Does this pathway play any role in other environmental stress? It would be nice to have a supplementary figure showing the resistance (or behavioral change) of ins-3 mutant worms in other stress conditions such as acute starvation (food-searching behavior or rate of motility), lifespan assay with pathogenic bacterial strain (Pseudomonas aeruginosa), etc.

<u>Response:</u> Thank you for this suggestion. We chose oxidative and thermal stress as representative environmental stressors because they elicit robust responses, as characterized in our previous publication (De Rosa et al, 2019). Following the reviewer's suggestion, we have now also included data on the resistance profile of *ins-3* mutants under starvation conditions (see Suppl Fig. 1, revised version). Consistent with our findings under oxidative and thermal stress, *ins-3* mutants also show an increased resistance to starvation stress compared to wild-type strains.

We believe that the inclusion of these three different environmental stressors provides a sufficient basis to conclude that *ins-3* plays a role in various environmental stress responses. Notably, tyramine-deficient mutants such as *tdc-1* and *tyra-3* exhibit greater

resistance to all three stressors (heat, oxidation, and starvation) compared to wild-type animals (De Rosa et al, 2019). While investigating the pathway's involvement in pathogen resistance would be interesting, this would fall outside the scope of the current study and in our opinion is a subject for future studies

2. Lines 133 - 137: The two sentences need more explanation. Heat stress for 30 min. in WT and heat stress for 10 min. in ins-3 mutants have the same outcome, i.e. nuclear accumulation of DAF-16. This isn't very clear and needs more explanation. The question that arises are 1) Why DAF-16 translocate to the nucleus in the WT condition? I would expect WT to respond differently compared to ins-3 mutant worms. 2) Why does DAF-16 take more time to move to the nucleus in WT worms? Is it because the initial flight response was converted into a cytoprotective response after prolonged exposure of 30 min.? If so, explain this. 3) Is there any other data that supports this explanation? If so, please include this data and explain appropriately.

Response: Thank you for pointing this out. In response to heat stress, DAF-16 nuclear translocation is faster in *ins-3* mutants than in the wild type. We have previously shown that wild-type animals require either 1) a higher intensity or 2) prolonged exposure to stress to induce DAF-16 nuclear translocation, compared to *tdc-1* and *tyra-3* mutants After 10 minutes of heat exposure, we observed significantly lower nuclear translocation of DAF-16 in wild-type animals compared to these mutants. However, after 30 minutes, the levels became similar, likely due to the saturation of the stress response (see Fig. 4. De Rosa et al, Nature, 2019). This finding led us to conclude that these mutants activate DAF-16 more readily in response to environmental stress than wild-type animals.

We find that after 10 minutes of heat stress, the amount of DAF-16 nuclear translocation is significantly higher in *ins-3* mutants compared to wild-type animals. This likely occurs because *ins-3* mutants do not require the downregulation of INS-3 secretion, which may be necessary for wild-type worms to induce DAF-16 nuclear translocation. In this regard, our results on the release of ins-3::venus reveal that there are tonic levels of INS-3 release in wild-type animals under basal conditions (Fig. 6, line 233, page 11 revised version). By 30 minutes of heat exposure, the levels of DAF-16 translocation in *ins-3* mutants and wild-type worms are similar (Fig. 8)., much more likely to what we previously observed with *tdc-1* and *tyra*-3 mutants (De Rosa et al, 2019).

For more clarity, we have removed the sentence referring to the 30-minute heat stress in this part of the manuscript, which we agree could be confusing at this point of the manuscript. The paragraph now reads as follows (line 119, page 7, revised version):

"ILP agonists can activate the DAF-2/IIS receptor which results in the phosphorylation of DAF-16 and its retention in the cytosol (Kenyon, 2010). To investigate whether intestinal

INS-3 systemically activates the DAF-2/IIS pathway, we examined the subcellular localization of the transcription factor DAF-16/FOXO using the translational Pdaf-16::DAF-16::GFP reporter. Under basal conditions, DAF-16 was predominantly localized in the cytoplasm in both wild-type and ins-3 null mutant backgrounds (Fig. S2). Following mild stress induction (10 min at 35°C), ins-3 mutants showed a significantly higher nuclear accumulation of DAF-16 compared to wild-type animals (Fig. 3A). This suggests that in ins-3 mutants, DAF-16 is more readily activated in response to stressors. Notably, the tissue-specific rescue of ins-3 in the intestine reduced nuclear DAF-16 accumulation (Fig. 3A). "

We believe this change improves clarity and better focuses the point on the increased nuclear translocation of DAF-16 observed in *ins-3* mutants.

3. Is the fluorescence intensity quantification experiment (Fig. 3B, 4, and 5) repeated 3 times independently? If not, at least repeat one more time to confirm the reproducibility.

Response: All fluorescence intensity quantification experiments were conducted with a minimum of three independent repetitions. The number of repetitions for each experiment has been clarified in the corresponding figure legends.

4. Figure 4: Representative worm images are not so convincing. Instead of having one worm in the field have many worms as shown below. If possible, apply this strategy to other images too.

Response: Following the reviewer's suggestion, we have included representative images of three different worms per condition in all the main figures of the manuscript.

5. Figure 5: The authors showed tyramine treatment increased ins-3::venus fluorescence intensity in coelomocytes without additional transcription of Pges-1::ins-3::venus. It will be nice, as well as recommended, to show that the fluorescence intensity in intestinal tissue decreases in response to the release of INS-3. Also, can the authors use tyra-3 mutant worms to show that ins-3::venus did not increase in the coelomocytes?

Response: This is an excellent point. We quantified *ins-3::venus* fluorescence in the intestines of animals under basal conditions and after tyramine exposure as suggested (see figure below). While there is a trend toward reduced fluorescence in tyramine-exposed animals, the differences are not statistically significant. We believe that detecting a decrease in fluorescence is challenging due to the already low baseline

levels. As mentioned in our response to Reviewers 2 and 3 we were only able to generate transgenic animals using low concentrations of the plasmid containing *ins-3::venus*. We hypothesize that *ins-3* overexpression may be deleterious to the animals, possibly due to overactivation of the DAF-2 pathway. This could explain why only animals with lower *ins-3* expression levels are viable. Our findings are supported by the observation that *ins-3* overexpression has been shown to shorten the lifespan of L1-arrested animals more than any other ILP (Zheng et al., 2018, JBC), suggesting a potential toxic effect of high *ins-3* levels. In contrast to the low fluorescence in intestinal cells, we can observe increased fluorescence in coelomocytes of animals treated with tyramine, as the released VENUS from multiple intestinal cells is concentrated in six coelomocytes. We have clarified the issues related to obtaining transgenics that overexpress *ins-3* in the intestine in the methods section (lines 598-602, page 23, revised version).

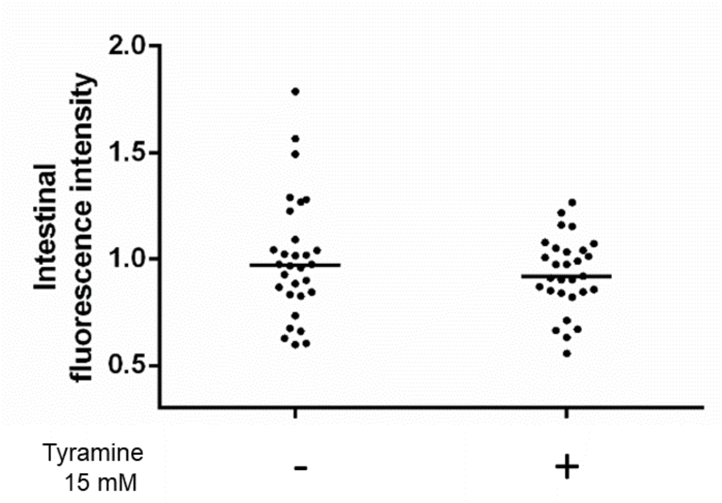

Additionally, following the reviewer's suggestion, we analyzed the release of *INS-3::VENUS* in a *tyra-3* mutant background. In support of our hypothesis, we find that a *tyra-3* mutation inhibits tyramine-triggered INS-3 release from the intestine (Fig.6, revised version). We believe this experiment, as suggested by the reviewer, is key to supporting our hypothesis regarding the role of the intestinal GPCR TYRA-3, and we thank the reviewer for this suggestion.

6. The model diagram (Figure 7B) can be improved by 1) adding a "?" to the unknown regions of the pathway (activation of TYRA-3 releasing the INS-3 vesicles), 2) Mark the signaling pathway that was previously found by citing the articles and 3) Mark the signaling pathway that is explained in this article.

Response: Following the reviewer's suggestions, we have updated the diagram to indicate which parts of the pathway were previously known (citing relevant papers),

which parts remain unknown, and which aspects are elucidated in this study. Additionally, as part of the experiments conducted for this revision, we demonstrated that tyramine induces IP3-dependent calcium release in the intestine, and this finding has been incorporated into the updated diagram.

7. What is the role of other tyramine receptors, such as ser-2, *tyra-2*, etc., in this pathway? It will be nice to see how mutants of other tyramine receptors behave under oxidative and thermal stress, at least as a supplemental figure.

Response: The role of other tyramine receptors, such as *ser-2*, *lgc-55*, and *tyra-2*, in this pathway was evaluated in our previous work (De Rosa et al., 2019). In that study, we analyzed the resistance of mutants for these receptors to oxidative and thermal stress (Figs. 3 and S5, De Rosa et al, 2019). Our findings revealed that TYRA-3 is the critical receptor for regulating the tyraminergic response to stress, as *tyra-3* mutants displayed resistance levels comparable to those of animals that cannot produce tyramine (*tdc-1* mutants). To further address the reviewer's comment, we find that TYRA-3 is the main receptor that mediates the high-frequency calcium waves in the intestine in response to exogenous tyramine. The reduction in high-frequency calcium transients in response to exogenous tyramine are similar in *tyra-3* vs a quadruple *tyra-3*, *ser-2*, *lgc-55*, *tyra-2* mutant (Fig S8, revised version). These data are consistent with TYRA-3 being the main receptor that mediates tyramine-induced intestinal calcium transients.

Minor:
Line 71 - Can you please mention the specific pair of neurons that release tyramine in this sentence? It will be helpful to know it right-a-way instead of going back to the citation.

Response: Thank you, done.

Line 119 - " ......promoter in a ins-3 null....." or "........an ins-3 null........."?

Response: Thank you, fixed.

Figure legend: When you mention "n=3", I understand that it is 3 experiments trials or biological replicates. It can be made clear by "n=3 experimental trials" or "n=40-50 worms per condition (in other figures)". For example: The authors have done this in figure 6A legend, "n=6-15 independent experiments".

Response: Thank you for pointing out the lack of clarity regarding the sample sizes. For certain experiments, typically stress resistance assays, we conducted 3-5 (or more)

independent experiments, evaluating 40-80 worms (or more) per condition per experiment. In these cases, the 'n' refers to the number of independent experiments, as shown by the data points in the figure (e.g., Figure 1A, revised version). We have clarified this in the figure legend with the following: "*Three independent experiments were performed (n=3). Each experiment included 60-80 animals per condition*".

In other cases, typically for fluorescence quantifications, we analyzed 40-80 animals, evenly distributed across three or more independent experiments (e.g., Figure 3A, revised version). For these experiments, we included the following in the figure legends: "*n= 45-50 animals per condition distributed across (three to four) independent experiments were evaluated*". We have clarified this point in each of the figure legends in the revised manuscript.

Figure 2A - Please mark the anterior-posterior directions of the worm and also use a dashed white line to outline the worm (similar to S4). Also, do this for all the figures.

Response: Done for all figures.

Figure 2 legend: Which graph uses a t-test? Please mention which graphs are analyzed using one-way ANOVA and which using t-test. Please follow this for all the figure legends.

Response: Done for all figure legends.

Figure 3A legend: "normalized to naïve animals", it is not completely understandable. What are naïve animals? If a normalization is done (heat shock animal/naïve animals) what is the proper label for the Y-axis in Fig 3A? Also, explain naïve animals in other figure legends.

Response: Thank you for bringing this to our attention. We apologize for the lack of clarity regarding the term "naïve animals." In the case of Figure 3A, we have clarified in the legend that the data are normalized to the wild-type values. We have also updated the Y-axis label to reflect this normalization. Additionally, we have replaced the term "naïve animals" in all figure legends, specifically clarifying the comparison point (e.g., basal, wild-type without tyramine, etc.), to ensure our meaning is clear

In the discussion, please discuss the role of other tyramine-producing cells such as uv1 cells near the vulva and gonadal sheath cells in stress response, if any?

Response: Thank you for this suggestion. In our previous work, we demonstrated through optogenetic stimulation and pharmacological silencing that activating RIM neurons and the subsequent release of tyramine is sufficient to inhibit the response to environmental stressors (De Rosa et al, Nature, 2019). Furthermore, RIM neuron activity has been strongly linked to the escape response, and we showed that its activity is suppressed when animals face stressors like oxidative stress, heat, or starvation (Donnelly et al, 2013, de Rosa et al, 2019). This supports the idea that tyraminergic signaling from RIM neurons plays a key role in driving a non-cell-autonomous stress response, which has been confirmed in subsequent studies by other labs (Ozbey et al, Cell Development 2020). While we cannot entirely rule out a marginal role for tyraminergic signaling from UV-1 cells in coordinating stress responses, the evidence strongly suggests that RIM activity is central to the nervous system's orchestration of stress responses. Following the reviewer's suggestion, we have included a sentence highlighting the expression of *tdc-1* (the gene encoding tyramine decarboxylase, responsible for tyramine synthesis) in other cells beyond RIM (Lines 317-321, page 14, discussion section, revised version), emphasizing that these neurons are the primary contributors to the tyraminergic coordination of the stress response:
Tyramine, considered the counterpart of adrenaline in C. elegans, is synthesized through the decarboxylation of the amino acid tyrosine by the enzyme TDC-1. This enzyme is expressed in the tyraminergic RIM neurons, octopaminergic RIC neurons, and neuroendocrine UV1 cells [33]. Despite its expression in these various cell types, we previously demonstrated that tyramine release specifically from RIM neurons during the flight response activates the TYRA-3 receptor in the intestine [32]

Line 410 - Please add a space between 35 and mm in "35mm plates". As well as follow a uniform writing style instead of "35-mm" (line 423, 426, etc.).

Response: Done. Thank you.

Line 414 - how many time the bacterial culture was concentrated

Response: We have now explained more carefully the process for cultured RNAi Bacteria in the Methods section. Fresh HT115 *E. coli* bacteria transformed with the L4440 empty vector or carrying the appropriate RNAi clone were grown in 2 mL of LB containing 100 mg/ml ampicillin at 37°C overnight. Grown bacteria were centrifuged 2 min at 5000 rpm and resuspended into 500 µL of LB. 75 µL of this resuspension were seeded in RNAi plates. These plates were kept overnight at 20˚C for RNAi induction.

Line 450 - please write a few lines about the plugin used for fluorescence intensity quantification.

Response: thank you. We have detailed the process for quantifying fluorescence intensity using the created FIJI Macro (Materials and methods line 476, page 19 revised version). Additionally, we have included the instructions for creating this Macro in (https://osf.io/wfgvs/).

Line 478 - please explain naïve condition.

Response: We have now avoided using the term naïve and specified the comparison condition/strain in each case.

Line 494 - "elt-2" should be in italics.

Response: Done. Thank you.

Line 525 - space between "30" and "mM" in "30mM".

Response: Done. Thank you.

General:
Check for "full stop" or "period" at the end of the sentences. It is missing in several places and make everything uniform throughout the manuscript. Please check the typos and punctuation throughout the manuscript. In some places, uniformity is missing, like "Scale bar, 50 µm" and in other places "Scale bar 50 µm", "Discussion", etc.

Response: Thank you for pointing these out. We have thoroughly reviewed the manuscript to correct these types of errors.

Reviewer #2: In this study by Veuthey et al., the authors highlight that a mutant form of the insulin-like peptide ins-3, acting as an agonist for the DAf-2 pathway, displays incresed resistance to heat and oxidative stress. Their research underscores the significance of tyramine secretion via tyraminergic neurons in facilitating the systemic impact of ins-3 release from the intestine, ultimately enhancing stress resilience by activating DAF-16 within the intestine of C. elegans.

This study builds upon their previous work, where they initially observed that neural stress hormones trigger the release of ILPs from the intestine, influencing stress responses. Their current focus on the ILP INS-3 reveals that reduced ins-3 levels or expression amplify DAF-16 activity in the intestine.

The major issue of this work is their overall conclusion, that tyramine-induced ins-3

release in the intestine leads to heat stress resistance via DAF-16 and HSF-1 activation, which is not supported by experimental evidence. In particular, the sequence of events as proposed in their model in Figure 7 lacks experimental evidence. While there is some evidence for DAF-16 to be involved in this response, there is no data at all regarding HSF-1. Another issue is that the study heavily relies on fluorescence intensity measurements and reporter strains, which are not sufficiently conclusive.

Response: We would like to thank the reviewer for the insightful comments. We agree that additional experiments were necessary to confirm the mechanistic pathway linking tyramine release to the stress response. We now have included genetic experiments, qPCR to validate the fluorescence experiments, and intestinal RNAi silencing experiments for this revision. In addition, we added intestinal calcium imaging experiments to show that tyramine activation of TYRA-3 induces strong calcium transients in the intestine. These new data strongly support a model where tyramine activates the Gq-coupled GPCR TYRA-3 in the intestine and increases calcium levels in IP3 dependent manner. TYRA-3 dependent activation and increase in calcium levels lead to the release of the ILP INS-3, which in turn activates DAF-2, inhibiting the phosphorylation of DAF-16 in various tissues. This inhibition of DAF-16 is responsible for the decreased resistance to environmental stress typically seen in animals with excessive tyraminergic activity.

Specific points:

Figure 3B and Figure 4: Confirm expression levels by Western blot using GFP antibody. A major shortcoming of this study is the lack of biochemical evidence by measuring protein expression using Western Blot analysis. Simply quantifying fluorescence intensity of hsp-16p::GFP and ins-3p::GFP is not sufficient here.

Response: We appreciate the suggestion to perform a Western blot to confirm protein expression levels of *hsp-16p::GFP* and *ins-3p::GFP*. However, we would like to highlight that in *C. elegans*, measuring GFP reporter fluorescence intensity is widely accepted as a proxy to assess protein expression. This approach has been used extensively in the field, including for insulin signaling, where the expression of *ins* peptides, transcription factors, and intracellular defense proteins such as DAF-16, HSFs, SOD-3, etc has been evaluated (Ritter et al, Genome Research 2013; Murphy et al, PNAS 2007; Lazaro-Pena et al, Elife 2023; De Rosa et al, Nature; 2019; Chauve et al, PLOS Biology, 2021; Sanchez-Blanco et al, 2011, PLOS Genetics and many others). Fluorescence-based methods allow us to analyze expression levels without the need to lyse the animals, which preserves the integrity of the tissue and allows measurements in living organisms.

However, to further address the reviewer's comments and provide additional support for our findings, we have conducted qPCR experiments. Our qPCR data show that *ins-3* mRNA levels decrease when animals are exposed to oxidation and heat, and increase when exposed to tyramine, which is consistent with our fluorescence results (Fig 4, revised Version).

Figure 5: the enhanced fluorescence intensity of the ges-1p::ins-3::Venus reporter is not a consequence of tyramine inducing an increase in ins-3 transcription. The ges-1p promoter is an intestine-specific promoter that overexpresses ins-3 in the intestine: is not under the control of the ins-3 endogenous promoter. This means that the conclusion they are drawing from this result cannot be accurate. To really confirm that ins-3 transcripts are not induced by tyramine, qPCR measurements of ins-3 mRNA is necessary. Moreover, it is unclear why the expression of ins-3::venus under control of ges-1p (an intestine-specific promoter) does not show any fluorescence in the intestine.

Response: We agree that it is important to verify that tyramine does not upregulate *ins-3::venus* via an effect on the *ges-1* promoter. To address this concern, we performed qPCR to detect *venus* transcripts (since these animals are not *ins-3* deficient, we considered it more appropriate to quantify *venus* expression than ins-3 transcripts). We analyzed animals under basal conditions and after exposure to 15 mM tyramine. As shown in Figure S10 of the revised version, exogenous tyramine did not increase *venus* transcript levels, suggesting that the observed increase in fluorescence in the coelomocytes is indeed due to enhanced secretion of *INS-3::Venus*, rather than increased transcription.

We also noted the low intestinal fluorescence in animals expressing ges-1p::ins-3::venus and encountered significant difficulty in generating these transgenic lines. We had to inject DNA at very low concentrations to obtain viable lines. Previously, we used a construct with the strong intestinal promoter *elt-2*, but were unable to generate transgenic animals. We hypothesize that overexpression of *ins-3* may be deleterious to the animals due to overactivation of the *daf-2* pathway. This could explain why only animals with lower levels of *ins-3* expression are viable. Supporting this idea, *ins-3* overexpression has been shown to shorten the lifespan of L1-arrested animals more than any other ILP (Zheng et al., 2018, JBC), which suggests a potential toxic effect of high INS-3 levels. We have explained this difficulty in the Materials and Methods section, specifying the DNA concentrations used to generate transgenic animals.

As we mentioned in our responses to reviewer #1, we believe that the concentration of VENUS released from the entire intestine into just six coelomocytes makes it feasible for us to detect increased fluorescence in these cells following exposure to tyramine.

Figure 6B: Figure 6B's findings that rescuing ins-3 in tyra-3 and tdc-1 mutants doesn't significantly impact stress resistance suggests that tyraminergic signaling likely has other targets contributing to stress resilience and that ins-3 is not the sole contributor that modulates stress survival. If it were, then the expectation is that the intestinal ins-3 rescue strain suppresses the increased stress resistance of the tyra-3,ins-3 and tdc-1,ins-3 double mutnats. Moreover, the correct statistical analysis comparing tdc-1,ins-3 and tyra-3,ins-3 double mutants with the rescued intestinal ins-3 to the same genetic background is also necessary here.

Response: We appreciate the reviewer's comment and apologize if we were not clear enough in the explanation of these results. The results presented in Figure 6B show that the intestinal rescue of *ins-3* can reduce the stress resistance of *ins-3* mutants, but only in strains without defects in tyraminergic signaling. In double mutants such as *tdc-1;ins-3* (which have a deficiency in tyramine synthesis) and *tyra-3;ins-3* (which lack the relevant tyramine receptor for the stress response), the intestinal *ins-3* rescue did not have a significant impact on stress resistance. These findings, along with the results from the original Figure S5 (now Figure S11, revised version), where we demonstrated that the intestinal expression of *ins-3* restores the deleterious effects of exogenous tyramine in *ins-3* mutants, but not in the *tyra-3;ins-3* double mutant, provide strong evidence that *ins-3* acts downstream of tyramine secretion and TYRA-3 activation. The sequence, as depicted in the schematic (Figure 8), is as follows: Tyramine Release (from RIM neurons) → Activation of TYRA-3 → Release of ins-3.

Following the reviewer´s suggestion, we have now included the correct statistical comparison between the double mutants (tdc-1;*ins-3* and *tyra-3*;*ins-3*) and the strains that express ins-3 in the intestine.

These experiments are now explained under results (Page 11-12, revised version):

"*To further investigate the crosstalk between tyramine and INS-3 in modulating the stress response, we examined the resistance of ins-3;tdc-1 and ins-3;tyra-3 double mutants. ins-3;tdc-1 and ins-3;tyra-3 double mutants did not show any further enhancement in stress resistance compared to tdc-1, ins-3 or tyra-3 single mutants (Fig 7B). This observation is consistent with the notion that tdc-1, tyra-3 and ins-3 genes act in the same pathway to regulate the environmental stress response. Unlike our observations in ins-3 null mutant backgrounds, the intestinal rescue of ins-3 in tyra-3;ins-3 and tdc-1;ins-3 backgrounds, did not impact the stress resistance (Fig 7B). Furthermore, exogenous tyramine failed to decrease stress resistance in the intestinal ins-3 rescue strain in tyra-3 null mutant background (Fig S11). These findings strongly support the hypothesis that INS-3 acts downstream of tyramine to modulate the stress response*"

Figure 7: The continued conclusion throughout the paper that the increased release of INS-3 inhibits DAF-16 and HSF-1 activity is unfounded. It is necessary to show that INS-3 acts through the DAF-2 signaling pathway in the intestine. One way to show this is by intestine specific knockdown of HSF-1 and DAF-16. Does intestine-specific knockdown of hsf-1 and daf-16 in the genetic background of ins-3, tdc-1 or tyra-3 mutants still lead to increased stress resistance? How does this alter expression of DAF-16 and HSF-1 targets? It will be necessary to measure transcripts or provide Western blot analysis of HSF-1 targets such as *hsp-70*. *hsp-16* is not a proxy for HSF1 activity as it is under control of both DAF-16 and HSF-1.
Moreover, the study's implication of the daf-2/ILS pathway's raises questions into potential effects of tyramine mutants on lifespan, a direction that could provide deeper insights into the mechanisms at play.

Response: We appreciate the reviewer for bringing this to our attention. We agree that our initial data on the involvement of the *daf-2* pathway were based on the analysis of DAF-16 localization and *hsp-16* expression, but we had not yet established a causal relationship between these phenomena and the increased stress resistance in *ins-3* mutants. Therefore, we decided to conduct genetic experiments to provide more direct evidence.

First, we analyzed the stress resistance of *daf-2* null mutants, which are well-known to be highly stress-resistant. We exposed wild-type animals, *ins-3* mutants, *daf-2* mutants, and *daf-2;ins-3* double mutants to heat and oxidative stress (Figure 3B, revised version). We applied more harsh stimuli (7 h, 35 °C and 3h 15 mM FeSO4, respectively) (De Rosa et al, Nature, 2019) because under the milder conditions regularly used in this manuscript, nearly 100% of *daf-2* mutants survived, which would have made it impossible to detect any additive effect in the double mutants due to a ceiling effect. Our results reveal that the absence of *ins-3* does not add to the enhanced stress resistance of *daf-2* mutants, strongly suggesting that the release of INS-3 modulates stress resistance through activation of DAF-2.

Additionally, we conducted genetic experiments with *daf-16* (Fig 3C, revised manuscript). As expected, *daf-16* mutants are more sensitive to heat and oxidative stress compared to wild-type animals, consistent with several reports (Deng et al, 2020https://doi.org/10.1534%2Fg3.120.401203; Lin XX, et al 2018, https://doi.org/10.1038/s41467-018-06624-0; Senchuk et al, 2018, https://doi.org/10.1371/journal.pgen.1007268). Importantly, *ins-3;daf-16* double mutants show stress resistance levels that are not significantly different from *daf-16* single mutants, revealing that the stress resistance in *ins-3* mutants is dependent on DAF-16 activation. These results complement our DAF-16 nuclear translocation experiments and provide conclusive evidence that INS-3 activates DAF-2 to inhibit DAF-16 activation.

Following the reviewer's suggestion, we also examined the importance of intestinal DAF-16 activity in the enhanced stress resistance of *ins-3* mutants. We crossed *ins-3* mutants with the VP303 strain, which allows for intestine-specific RNAi (Espelt MV, Estevez AY et al (2005), J Gen Physiol, http://www.jgp.org/cgi/doi/10.1085/jgp.200509355; Wu et al (2021), Cell Reports https://doi.org/10.1016%2Fj.celrep.2021.109529). We found that, although the stress resistance in *ins-3* mutants with intestine-specific *daf-16* knockdown is higher than in the wild-type background, it is significantly lower than in *ins-3* mutants alone (Figure 3D). This strongly suggests that the inhibition of *ins-3* release activates DAF-16 in the intestine, but that this activation only partially contributes to increased stress resistance. These data indicate that nuclear translocation of DAF-16 in other tissues is also important (as represented in the final schematic).

We also performed genetic experiments with *hsf-1* mutants and found that *hsf-1* mutants are no more sensitive to heat and oxidative stress than wild-type animals (Fig S5). Comparing these results to those for *daf-16*, we conclude that DAF-16 is the more relevant transcription factor for stress resistance in response to the stimuli presented here. Moreover, although we observed a reduction in oxidative stress resistance of *ins-3;hsf-1* double mutants compared to *ins-3* animals, this was not the case for heat, where the loss-of-function of *hsf-1* did not impact the resistance of *ins-3* mutants (Fig. S5A). Finally, intestinal knockdown of *hsf-1* by RNAi did not affect the stress resistance of *ins-3* mutants. These data suggest that the role of *hsf-1* in the tyramine-*ins-3-daf-2* stress response pathway is minor.

Based on these experiments, we were able to reach this conclusion, and we thank the reviewer for helping us improve our work. Lastly, *hsp-16* levels (which, as the reviewer correctly points out, are regulated by both *hsf-1* and *daf-16*) were validated by qPCR and have been included in Figure S4B of the revised version. We have also corrected the sentence in the results referring that *hsp-16.2* is regulated by both HSF-1 and DAF-16 and include references (lines 156-158 and 165-167, Page 8, revised version)

Reviewer #3: Veythey et al manuscript on neurohormone tyramine that stimulates insulin-like peptide from intestine to modulate systemic stress in C. elegans

Strengths of this paper is it adds to the studies that investigate the role insulin-like peptides have in stress responses and the signaling pathway from gut and signaling pathway; C. elegans is a good system to investigate this.

The following need to be adressed (most minor issues)
1. Methodology: The heatshock protocol involved putting plates, sealed with parafilm, placed in a ziplock bag, and immersed in waterbath for 20hrs. Authors likely did this to

ensure uniform temperature exposure to the worms. My concern is that the O2 levels could fluctuate in this environment. Unsure if they have thought of hypoxia as a compounding issue. Also within methods, the strain list (line 379)- may want to add references for each strain that was developed elsewhere.

Response: We apologize for not being clear enough in the description of the methodology for these experiments. The heat shock exposure lasted only 4 hours, after which we removed the plates from the water bath, unsealed them from the parafilm and ziplock bags, and allowed the worms to recover at 20°C for 20 hours before quantifying survival. Therefore, the period with potentially low oxygen levels was limited to just 4 hours. When we optimized this protocol (De Rosa et al, Nature, 2019, https://www.nature.com/articles/s41586-019-1524-5), which was adapted from previous reports of other labs (Phralad et al; Science 2008 https://doi.org/10.1126/science.1156093), we measured the survival of animals after 4 hours in sealed plates with parafilm and ziplock bags a 20°C, and found a 100% survival rate. While we cannot completely rule out a contribution of hypoxia to our heat stress results, we believe that any such effect would be very minor. We have clarified this point in the Materials and Methods section (Page 19, lines 455-458, Revised Version)
"*Thermotolerance assays were performed as described (De Rosa et al., 2019). For each strain, four 35 mm NGM agar plates containing 20 animals (with or without 15 mM exogenous tyramine) were incubated at 35°C for 4 h. To ensure proper heat transfer, 6-mm-thick NGM agar plates were used. Animals were synchronized as L4s and used 14 h later. Plates were sealed with Parafilm in zip-lock bags and immersed in a 35°C water bath for 4 h. After that period, the plates were taken out from the water bath and the zip-lock bags and parafilm were removed. Surviving animals were counted after a 20 h recovery period at 20°C. For all assays, animals were scored as dead if they failed to respond to prodding with a platinum-wire pick to the nose of the animal".*

Results:
2. Line 443 period at end of sentence missing and paragraph return typo? Line 456 period at end of sentence missing (check for typos throughout the manuscript)

Response Thank you very much. We have made those corrections and also fixed other typographical errors throughout the manuscript

3. Figure 2A state the larval stage of the animal 4.

Response: Thank you. In the figure legend, we have now stated that the animal shown is in the L4 larval stage.

Figure 2B - was RTPCR done to ensure that the PrGEF-1 (neurons) did indeed induce expression of ins-3. That is, the non-rescue isn't because of the ins-3 not being properly expressed in the neurons but that the neuronal ins-3 does not have a role in the survival.

Response: Thank you for this suggestion. We performed qPCR on animals transformed with *Prgef-1::ins-3* DNA. Unlike the *ins-3* mutants, we detected *ins-3* transcripts in these transgenic animals, strongly suggesting that *ins-3* is indeed expressed. However, despite this neuronal expression of the *Prgef-1::ins-3* transgene in the *ins-3* mutant background does not rescue their sensitivity to environmental stress, indicating that neuronal *ins-3* does not play a role in stress survival. We show these data in table S1 (pasted below) and refer to this control in the Materials and Methods section (lines 589-592, page 23 revised version).

	ins-3 null mutant		*ins-3; Prgef-1::ins-3* (neuronal rescue)	
	Insulin-3	*actin*	*Insulin-3*	*actin*
CT	44,53 ± 3,30	14,80 ± 1,44	20,20 ± 1,24	19,33 ± 0,87
ΔCT (CT *insulin-3*-CT *actin*)	29,73 ± 3,52		2,2 ± 1,74	

Values of Ct above 35 are below the detection limit and are considered as background noise.

5. Line 145/146 - add a reference that shows hsp-16.2 is an HSF-1 effector gene and refer to hsp-16.2 as such (instead of hsp-16).

Response: Thank you. We have made the necessary corrections and included the relevant references. Additionally, we revised the paragraph to highlight that *hsp-16.2*

depends not only on HSF-1, as extensively reported, but that there are also some reports showing its dependence on DAF-16 (Lines 155-160, page 8, Revised version).

6. Fig S3- it does help having the reporter noted in the figure (like Fig S1) and not just legend so perhaps add "ins-4 expression" to top of figure

Response: Thank you. Done

7. Fig 4 and sentence on line 175-177- it looks like ins-3::GFP is expressed in the posterior part of the intestine when exposed to exogenous tyramine. Add "posterior" to further specify (levels do not seem throughout the intestine). Has higher magnification of specific areas of the intestine been analyzed to know which intestinal cells have the expression of ins-3::GFP in response to tyramine.

Response: The reviewer is correct in noting that the expression of ins-3::GFP is notably increased in the posterior part of the intestine upon exposure to exogenous tyramine, with only faint expression observed throughout much of the rest of the intestine. This pattern correlates with the expression of the tyramine receptor TYRA-3, which is known to be more pronounced in this region (De Rosa et al., Nature 2019).
It is possible that the higher expression of TYRA-3 in this part of the intestine may drive increased transcription of INS-3 in these cells. Although it would be interesting to further differentiate the roles of various intestinal cell types in the neural control of stress responses, this is beyond the scope of our current study and is planned for future research. We updated the figure legend to specify that the measurements correspond to the posterior third of the intestine, to clarify the region of interest in our analysis.

8. In the text that states "young adult" can the age be clarified and if gravid or not (1 day old vs non gravid after molting); just for clarity.

Response: Thank you for pointing this out. For the microscopy experiments, we clarify that "young adults" refers to animals that are post-last molt and have not yet become gravid. This specification is made to ensure that expression levels are not influenced by the presence of eggs, thereby providing a more accurate quantification.

9. Tyramine triggering INS-3 release - why wasn't INS-3 translational reporter with endogenous promotor (e.g. CRISPR strain) used; The expression level of Pges-1::ins-3::venus seems pretty low given it is driven by Pges-1. Also can authors clarify if the venus marker was used to minimize autofluorescent issue from the intestine and that autofluorescence not observed.

Response: Our hypothesis is that tyramine mediates intestinal release of INS-3, which is why we utilized an intestinal-specific promoter. Using an endogenous promoter would have made it difficult to discern whether the release was from the intestine or neurons, where INS-3 is also expressed.

The reviewer is correct in noting the low intestinal fluorescence in animals expressing Pges-1::ins-3::venus. As mentioned in the responses to the previous reviewers, we faced significant challenges in generating these transgenic lines. To obtain viable lines, we had to inject DNA at very low concentrations. We also attempted to use the strong intestinal promoter elt-2 but were unable to produce transgenic animals. We speculate that high levels of INS-3 expression may be detrimental to the animals, as overexpression has been shown to shorten the lifespan of L1-arrested animals (Zheng et al., 2018, JBC). We believe that only strains with very low expression of INS-3::Venus were viable. This low expression makes it very challenging to detect and quantify the fluorescence in the intestine. Quantification in coelomocytes is more straightforward, as these cells likely concentrate Venus secreted from multiple intestinal cells.

Regarding the choice of Venus, we used this marker because it is resistant to quenching in low pH environments and has been effectively used to monitor neuropeptide secretion from dense core vesicles in *C. elegans* (10.1038/nn1810; doi: 10.1534/genetics.113.158568; doi: 10.1038/nbt0102-87). This explanation has been added to the Methods section (lines 557-560, page 22, revised version) to clarify why Venus was chosen over GFP.
