# Supplementary material for: The neurohormone tyramine stimulates the secretion of an insulin-like peptide from the Caenorhabditis elegans intestine to modulate the systemic stress response
Source: PLoS Biol. 2025 Jan 28;23(1):e3002997. doi: 10.1371/journal.pbio.3002997 (PMC11774402; doi:10.1371/journal.pbio.3002997)
Supplement: Attachment — Submitted filename: Rerebuttal.pdf [file pbio.3002997.s019.pdf]

We would like to thank the reviewers once again for the comments and constructive feedback. We are happy to hear that all reviewers appreciate the improved manuscript. Reviewers 1 and 3 raised no further issues. Reviewer 2 raised some minor, but valid points, that we can easily address by textual clarifications. See below

Reviewers 1 and 3 have no additional comments.

Reviewer 2 raised two minor concerns (Reviewer comments are in blue; response is in black):

While the authors have addressed all previous comments and the manuscript has significantly improved,

Thank you for helping us improve the manuscript.

1. I still have some concerns. Firstly, although the hsf-1(sy441) mutant has been demonstrated to be sensitive to heat shock in numerous prior studies, the heat-shock conditions used here (exposing C. elegans to 35°C for only 4 hours) may not be sufficient to observe this sensitivity. If the authors wish to claim that the hsf-1 mutant is not sensitive to heat shock and that HSF-1 plays only a minor role in the modulation of stress responses by INS-3, I recommend conducting a heat-shock experiment using hsf-1 RNAi (knockdown at the L2 stage). Another issue with this is that there has been a lot of controversy with regards to the hsf-1(sy441) mutant. Some labs observed the reduced thermotolerance of this mutant during heat shock conditions, whereas others observed increased heat stress resistance that is age-dependent (Morton et al, 2013; Golden et al, 2020, and Kovacs et al., Aging Cell 2024). This should be considered in the authors response and revised manuscript.

We appreciate the reviewer's comments. The reviewer is correct in noting that *hsf-1(sy441)* mutants have previously been demonstrated to be sensitive to heat shock under specific experimental conditions, such as exposure of adult animals to 35°C for 9 hours (*Prahlad et al.*, 2008), 15 hours at 32.5°C (*Finger et al.*, 2021), or day 6 adults exposed to 38°C for several days (*Steinkraus et al.*, 2008). The primary focus of our study, however, is not to analyze the role of HSF-1 in thermotolerance itself, but rather to determine whether HSF-1 mediates the effects of INS-3 on stress responses. To address this, we employed the same conditions used to assess stress resistance in *ins-3* mutants. Specifically, we exposed day 1 adult animals to 35°C for 4 hours. Under these conditions, we found that *hsf-1(sy441)* mutants exhibit greater thermotolerance than wild-type animals. Additionally, we observed no significant differences in thermotolerance between *ins-3* single mutants and *ins-3; hsf-1* double mutants (Fig. S5). As the reviewer points out the conflicting results of *hsf-1* in thermotolerance are most likely due to the different assay conditions. Our observations are consistent with a recent publication where they use similar conditions to ours (day 1 adults exposed to 35°C for 5 hours, compared to our use 35°C for 4 hours) and show that both *hsf-1(sy441)* mutants and RNAi-mediated knockdown of *hsf-1* exhibit increased thermotolerance compared to wild-type animals (Kovacs et al, 2024).

To avoid confusion, and acknowledge the role of HSF-1 in thermotolerance, we revised the text accordingly (Lines 164-180, Pages 8-9, revised version):

"Since *hsp-16.2* expression is regulated by both DAF-16 and HSF-1, we performed genetic experiments using the *hsf-1(sy441*) loss-of-function mutant. Animals were exposed to the same oxidative- (1 hour in 15 mM $FeSO_4$) and heat-stress (4 hours at 35°C) conditions under which *ins-3* mutants showed increased resistance. Under these conditions, *hsf-1(sy441)* mutants did not exhibit increased sensitivity to oxidative stress and were even more resistant to heat stress compared to wild-type animals (Fig. S5A). Our results are consistent with recent studies using similar heat stress conditions and developmental stages as those used in our study (Kovacs et al., 2024). While we observed a reduction in oxidative stress resistance in

ins-3;hsf-1 double mutants compared to *ins-3* single mutants, this was not observed under heat stress conditions. In fact, the loss of *hsf-1* function did not affect the increased heat resistance of *ins-3* mutants (Fig. S5A). These results suggest that DAF-16 activation appears to be the primary effector driving the enhanced stress resistance observed in these mutants. Our experiments, however, do not completely exclude a role for HSF-1 in modulating the responses to harsher stress conditions since *hsf-1* has been shown to play a critical role in stress resistance under prolonged stress conditions (e.g., thermotolerance after 9 hours at 35°C (Prahlad et al., 2008) or oxidative stress after 24 hours of exposure to 200 mM paraquat (Finger et al, 2021),

2. Secondly, I disagree with the assertion that measuring GFP fluorescence intensity as a proxy for protein expression in C. elegans. While measuring GFP fluorescence intensity is indeed accepted in the C. elegans field, it is not as rigorous and accurate as Western blot analysis. Indeed, many research papers utilize Western blotting with specific antibodies to quantify protein expression also in C. elegans (e.g. Tian et al., Cell 2016, Charmpilas et al, Cell Reports 2024).

We agree with the reviewer that Western blot experiments with antibodies raised against the endogenous protein are the gold standard for measuring total protein levels. Arguably, the western blot experiments against transgenic GFP reporters or GFP fusion proteins are still proxies for endogenous protein levels. While the most common approach in *C. elegans* is to measure fluorescence levels, some studies also use western blots. In these studies, western blot quantification has all been consistent with the fluorescence data indicating that western blot experiments are unlikely to provide any new information. Fluorescence levels are preferred over western blots on whole animal extracts since they also provide cellular resolution specifically in the intestine Since INS-3 antibodies are not available we felt that qPCR experiments would provide a better readout for the endogenous expression levels. The changes in fluorescence of the transcriptional reporters we observe are consistent with the qPCR experiments. In addition, the genetic experiments and the DAF-16 nuclear translocation all strongly support our model that DAF-16 is the relevant transcription factor in the modulation of stress response by *ins-3*.
